# Investigating gut permeability in neonatal calves with diarrhea: A case-control study

**DOI:** 10.3168/jdsc.2024-0709

**Published:** 2025-03-03

**Authors:** Luiza S. Zakia, Diego E. Gomez, Michael A. Steele, Peter D. Constable, Stephen J. LeBlanc, David L. Renaud

**Affiliations:** 1 Department of Population Medicine, Ontario Veterinary College, University of Guelph, Guelph ON N1G 2W1; 2 Department of Clinical Studies, Ontario Veterinary College, University of Guelph, Guelph ON N1G 2W1; 3 Department of Animal Biosciences, Animal Science and Nutrition, University of Guelph, Guelph, Ontario, Canada N1G 1Y2; 4 College of Veterinary Medicine, University of Illinois, Urbana, IL 61802

## Abstract

The objective of this case-control study was to assess gut permeability, measured through Cr-EDTA recovery, in healthy and diarrheic neonatal calves. The study was conducted at a commercial calf-rearing facility, where fecal consistency was monitored twice daily. Calves were categorized as diarrheic if they had runny or watery feces, whereas those with normal fecal consistency, neutrophil count, and physical exam findings were considered healthy controls. Gut permeability assessment (Cr-EDTA: 0.1 g/kg BW administered orally 2 h after milk feeding) and blood bacterial culture were performed 24 h after onset of diarrhea. Plasma Cr concentration was determined using inductively coupled plasma mass spectrometry. Wilcoxon signed-rank test and Kruskal-Wallis tests, followed by Dunn's post hoc test, were used to compare Cr concentrations between groups. The study included 12 healthy calves and 11 diarrheic calves, with 5 diarrheic calves having bacteremia. Diarrheic calves had greater median (interquartile range) plasma Cr concentrations than healthy calves at 2 h (1.76 [0.92–2.34] mg/L vs. 0.59 [0.48–1.19] mg/L) and 4 h (2.07 [1.57–2.51] mg/L vs. 0.92 [0.77–1.66] mg/L) postadministration, respectively. Both bacteremic and nonbacteremic calves with diarrhea had greater plasma Cr concentrations at 2 h compared with healthy calves (bacteremic: 1.96 [1.76–2.03] mg/L; nonbacteremic: 1.42 [0.78–2.34] mg/L), but there was no difference between bacteremic and nonbacteremic calves. At 4 h postadministration, both bacteremic (2.00 [1.67–2.07] mg/L) and nonbacteremic (2.45 [1.57–3.66] mg/L) diarrheic calves had greater plasma Cr concentrations than healthy calves, with no difference observed between bacteremic and nonbacteremic groups. This study suggests an association between diarrhea and increased gut permeability in neonatal calves. Further studies are required to compare gut permeability in a larger cohort of bacteremic and nonbacteremic diarrheic neonatal calves.

## Highlights

• Diarrhea in calves is associated with higher plasma Cr concentrations.
• Gut permeability was similar between bacteremic and nonbacteremic diarrheic calves.
• Gut permeability can be accessed by Cr-EDTA in neonatal diarrheic calves.

## Graphical Abstract

**Figure:**
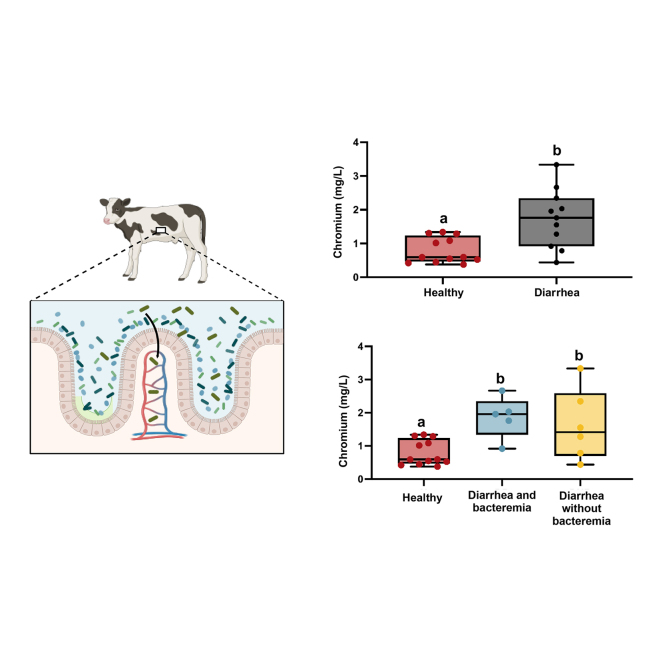
**Summary:** This case-control study assessed gut permeability in healthy and diarrheic neonatal calves. Calves were categorized as diarrheic if they had runny or watery feces, whereas those with normal fecal consistency, neutrophil count, and physical exam findings were considered healthy. Gut permeability and bacteremia were assessed 24 hours after diarrhea onset. Twelve healthy calves and 11 diarrheic calves were included, with 5 of the diarrheic calves being bacteremic. Diarrheic calves had higher plasma chromium (Cr) concentrations 2 and 4 hours after Cr-ethylenediaminotetraacetate (EDTA) administration compared with healthy calves. Both bacteremic and nonbacteremic calves with diarrhea had higher plasma Cr concentrations at 2 hours than healthy calves, with no difference detected between bacteremic and nonbacteremic. The results suggest that gut permeability is increased in diarrheic calves regardless of bacteremia. Labels a and b in the figure indicate significant differences between groups (P < 0.05). Created with BioRender.com.

Neonatal calf diarrhea (**NCD**) is a major concern for the dairy industry, affecting up to 50% of newborn calves (USDA, 2021). In addition, the neonatal period is crucial for calf health, with diseases during this period carrying long-term productivity consequences, such as decreased reproduction and milk production (Abuelo et al., 2021). Antimicrobial drugs (**AMD**) are currently recommended for treating and preventing bacteremia in critically ill diarrheic calves (Constable, 2004). The proliferation of pathogenic bacteria in the small intestine and bacteria translocation to the bloodstream is currently an extensively used hypothesis as a rationale for using AMD in NCD (Constable, 2004). However, these recommendations are based on expert opinion and are not evidence based.

The mechanism of development of bacteremia in diarrheic calves is not well understood. It is currently hypothesized that diarrhea-induced bacteremia in calves stems from increased gut permeability due to morphologic damage to the intestinal epithelium and mucosa, which leads to bacteria entering the bloodstream (Constable, 2004). Although translocation of *Escherichia coli* through the intestinal layers has been demonstrated in newborn calves (Corley et al., 1977), the hypothesis that increased gut permeability due to diarrhea allows enteric bacteria to reach the bloodstream has not been well demonstrated in NCD. Therefore, further understanding of the pathogenesis of bacteremia in NCD is needed.

One study demonstrated that the absorption of lactulose (marker of gut permeability) in calves that developed diarrhea between d 7 and 14 of age was increased since the day they were born, compared with those that remained healthy (Araujo et al., 2015). Although this suggests that gut permeability is increased in diarrheic calves, the study did not establish temporality and causality, leaving the causal association speculative. Further, another study showed increased gut permeability in calves with experimentally induced diarrhea 7 and 14 d after *Cryptosporidium parvum* inoculation (Klein et al., 2008). However, the impact of naturally occurring diarrhea on gut barrier function and the potential translocation of bacteria to the bloodstream has not yet been evaluated. Therefore, the objectives of this study were to investigate gut permeability in calves with diarrhea and to explore the possible association of increased permeability with bacteremia. We hypothesized that gut permeability is increased in calves with diarrhea compared with healthy calves, and more so in diarrheic calves that become bacteremic compared with those that do not.

This case-control study was approved by the University of Guelph Animal Care Committee (Animal Use Protocol number 4984). The study protocol was published online before the beginning of data collection (Zakia et al., 2023). The study was conducted at a calf-raising facility in Wellington County, Ontario, Canada, from June to August 2023. This facility was chosen due to its proximity to the University of Guelph, its intensive monitoring capabilities, its compliance with the Veal Code of Practice requirements, and for receiving calves from multiple dairy farms in Southwestern Ontario. Each room in the facility contains 64 individual stalls, each measuring 1 m × 1 m, with slatted rubber flooring. Calves were moved in and out of the rooms in groups, with all calves in a room arriving and leaving simultaneously. During the first week after arrival, calves were fed 2.5 L of the same milk replacer twice daily (total of 5 L/d) at 40°C and 130 g of powder per L (26% crude milk protein, 20% fat, 44% lactose, 4.71 Mcal of ME/kg). Calves had ad libitum access to water and texturized calf starter (18% CP, 26.5% starch, 17.3% NDF, 3.6% crude fat, and 5.6% ash; custom mix, Wallenstein Feed and Supply). Calves from 2 rooms (n = 128) were eligible to be included in this study.

The variables included in the physical exam were BCS, attitude, posture, suckle reflex, skin tent, the color and moistness of the mucous membranes, capillary refill time, scleral injection, presence of enophthalmos, heart rate, respiratory rate, respiratory effort, rectal temperature, navel score, and joint abnormalities. The first author (LSZ), a board-certified large animal internist, performed all the fecal scorings and physical exams. For gut permeability testing and data analysis, LSZ was not blinded to whether the calves had diarrhea.

Complete blood cell count was performed using Advia 2120i Hematology Analyzer (Siemens Healthineers, Erlangen, Germany) within 3 h of sample collection; samples were kept refrigerated at approximately 4°C from collection until analysis (Warren et al., 2013).

Starting the day after arrival, fecal consistency was evaluated following digital stimulation twice daily (0700 and 1700 h) as outlined by Larson et al. (1977). Calves were considered diarrheic and included as cases if they had runny or watery feces (fecal score 2 and 3, respectively). Calves were considered healthy and included as controls if they had normal or soft feces (fecal score 0 and 1, respectively), neutrophil count, and physical exam. Healthy calves were evaluated every 12 h and were only included in this study as controls if they remained healthy (normal fecal scoring and physical exam) for at least 24 h. Controls were matched based on the day of arrival at the facility (±1 d) to ensure controls and cases were of similar age. One control was chosen for every case included. Initially, we planned to do the gut permeability test on d 9 after the calves arrived at the facility (Zakia et al., 2023); however, due to the high incidence of diarrhea in the first few days after arrival, gut permeability was assessed on d 4 or 5 after arrival on both cases and controls.

Blood was collected for aerobic and anaerobic blood culture from diarrheic calves 24 h after the onset of diarrhea. Blood collection was performed aseptically as follows: an area of approximately 10 cm × 10 cm was clipped over the jugular vein, then the area was scrubbed with chlorhexidine gluconate 4% (BD E-Z Scrub, Becton, Dickinson and Co.) for 5 min, followed by five 70% alcohol swipes, followed by five 5% chlorhexidine gluconate tinted swipes. Blood collection was performed using a partially evacuated tube adaptor, and 10 mL of blood was collected into a BD BACTEC Standard Aerobic medium (Becton, Dickinson and Co.). Blood cultures were processed by the bacteriology laboratory of the Animal Health Laboratory, University of Guelph (Guelph, ON, Canada), using their standard operating procedure. Briefly, blood culture vials were incubated for 24 h at 35°C. After incubation, one drop of medium was plated on blood agar (**BA**), MacConkey (**MAC**), brucella, and phenylethyl alcohol (**PEA**) agar plates. Blood agar plates were incubated at 35°C with 5% CO_2_, whereas MAC plates were incubated at the same temperature in atmospheric air. Brucella and PEA plates were incubated at 37°C anaerobically with 5% CO_2_, 10% H_2_, and 85% N_2_. Plates were checked for bacterial growth at 24 and 48 h. When there was bacterial growth, individual colonies were smeared onto stainless-steel target plates and covered by α-cyano-4-hydroxycinnamic acid. The bacterial identification was made using the MALDI Biotyper Sirius system (Bruker Daltonics Inc., Bremen, Germany). In addition, blood culture vials were incubated for 5 d, and on the fifth day, aerobic culture using BA and MAC plates was repeated. With regard to fecal pathogen identification, fecal samples were collected directly from the rectum following digital stimulation at the onset of diarrhea and submitted to the Animal Health Laboratory for testing, which included *Cryptosporidium* spp. PCR, rotavirus A and B PCR, coronavirus PCR, and *E. coli* enterotoxigenic genotyping. Fecal samples were also collected 24 and 48 h after the onset of diarrhea, and a pool of the 3 samples was submitted for *Salmonella* spp. PCR to enhance the sensitivity of the test due to intermittent shedding of this pathogen (Mohler et al., 2009).

Gut permeability was evaluated using Cr-EDTA 24 h after the onset of diarrhea and standardized to be conducted on d 4 or 5 after arrival at the facility, ensuring similar ages for both diarrheic and healthy calves. The marker Cr-EDTA (Sigma-Aldrich) was administered orally (0.1 g/kg BW) via a 60-mL slip-tip syringe 2 h after the morning milk feeding. All the calves included in the study drank the morning milk. A liquid solution was prepared in advance using 66.7 g of Na_2_-EDTA, 47.7 g of CrCl_3_, and 1.937 g of CaCl_2_ per liter of distilled water. Following Cr-EDTA, 60 mL of water was administered via a syringe to ensure the calf swallowed the marker. Blood samples (10 mL) from the jugular vein were collected into a lithium-heparin tube (BD Vacutainer, Becton, Dickinson and Co.) before Cr-EDTA administration and 2 and 4 h later. The samples were centrifuged at 1,500 × *g* for 15 min at room temperature. The plasma was then aliquoted and frozen at −20°C until analysis. Plasma Cr concentration was determined by inductively coupled plasma mass spectrometry at the Water Quality Centre at Trent University.

Statistical analyses were conducted in STATA 18 SE (StataCorp LLC), and graphs were generated using GraphPad Prism 10 (GraphPad Software LLC). The sample size calculation for the gut permeability test was based on the previously reported serum Cr concentration of 74 µg/mL in healthy calves 2 h after oral administration and the standard deviation of 18 µg/mL (Cangiano et al., 2022). A 30% difference (22 µg/mL) was set as the expected difference between groups (healthy vs. diarrhea), considering that a 30% increase in Cr concentration would represent a clinically meaningful increase in gut permeability. The α level was set at 0.05 and the power at 0.8. A total of 24 animals (12 per group) were required for this study. Calves were excluded if they were extreme outliers (i.e., Cr measurements were biologically implausible). Chromium concentration was compared among groups using Wilcoxon signed-rank test, and Kruskal-Wallis test, followed by Dunn's post hoc test. Area under the curve was calculated for 4 h (**AUC_4_**) using the linear trapezoidal method. In addition, a linear regression model was built to evaluate the possible confounding effect of BW on arrival, room, plasma protein concentration, and hydration status (categorized as normal vs. abnormal) on Cr concentration. Confounding was set as a change greater than 25% on the coefficient of diarrhea status (diarrheic vs. healthy) in the linear regression models. Because the exact age of the calves was unknown, we used BW as a proxy for age. The outcome was the Cr concentration 2 h after the administration of the marker. Explanatory variables included BW on arrival, diarrhea status (diarrhea vs. healthy), and bacteremia status (bacteremia vs. no bacteremia). Linearity of predictors was assessed using a Lowess curve. Nonlinear predictor variables were categorized by quartile. Model fit was assessed by evaluating the normality (standardized residuals distribution and Shapiro-Wilk test) and homogeneity of variance (distribution of standardized residuals and Cook-Weisberg test). Leverage, Cook's D, and DFBETA plots were created to evaluate the leverage and influence of observations on the model and variables, respectively. In case of heteroscedasticity, a robust linear regression model was run to obtain reliable estimates for the parameters despite the presence of outliers.

Twelve healthy calves and 11 calves with diarrhea were included in this study. A 12th diarrheic calf had been included but was an extreme outlier, and therefore, was excluded from the study. One calf was female, and 22 were male. Nine calves were Holstein crossbreds and 14 were Holstein. Of the 11 calves with diarrhea, only 2 were from room 2, whereas 8 of the 12 healthy calves were from that room. Among diarrheic calves, 5 were bacteremic, and 6 were nonbacteremic. The bacteria isolated from the blood cultures are in Table 1. None of the calves were positive for coronavirus, *Salmonella* spp., or *E. coli* genotypes Sta, F5 (K99), and F41. Ten calves were positive for rotavirus A, and one for rotavirus B. Five calves were positive for *Cryptosporidium* spp. (Table 1).

**Table 1:** Blood culture results and fecal pathogen testing results from diarrheic neonatal calves; blood culture was not performed on healthy calves

Calf	Blood culture	Coronavirus fecal PCR	*Cryptosporidium* spp. fecal PCR	ETEC genotype (Sta, F5 [K99], and F41)	Rotavirus A fecal PCR	Rotavirus B fecal PCR	*Salmonella* spp. fecal PCR
1	—	Negative	Positive	Negative	Negative	Negative	Negative
2	—	Negative	Negative	Negative	Positive	Negative	Negative
3	—	Negative	Negative	Negative	Positive	Negative	Negative
4	—	Negative	Negative	Negative	Positive	Negative	Negative
5	—	Negative	Positive	Negative	Positive	Negative	Negative
6	—	Negative	Positive	Negative	Positive	Negative	Negative
7	*Streptococcus dysgalactiae* and *Pasteurella* spp.	Negative	Negative	Negative	Positive	Negative	Negative
8	*Staphylococcus simulans*	Negative	Negative	Negative	Positive	Negative	Negative
9	*Staphylococcus chromogenes* and *Enterococcus faecalis*	Negative	Negative	Negative	Positive	Negative	Negative
10	*Clostridium tertium*	Negative	Positive	Negative	Positive	Positive	Negative
11	*Streptococcus ruminantium*	Negative	Positive	Negative	Positive	Negative	Negative

All calves tested had plasma Cr concentrations of less than 0.04 mg/L before Cr-EDTA administration. There was no difference (*P* = 0.2139) in Cr concentration between healthy and diarrheic calves before Cr-EDTA administration. Diarrheic calves had greater median (interquartile range) plasma Cr concentrations than healthy calves 2 h (1.76 [0.92–2.34] mg/L vs. 0.59 [0.48–1.19] mg/L; *P* = 0.007) and 4 h (2.07 [1.57–2.51] mg/L vs. 0.92 [0.77–1.66] mg/L; *P* = 0.02) after Cr administration (Figure 1). Among calves with diarrhea, plasma Cr concentration at 2 h was greater in both bacteremic (1.96 [1.76–2.03] mg/L; *P* = 0.004) and nonbacteremic (1.42 [0.78–2.34] mg/L; *P* = 0.04) calves than healthy calves, but no difference was detected between bacteremic and nonbacteremic calves (*P* = 0.20; Figure 2). At 4 h after dosing, plasma Cr concentration tended to be greater in bacteremic (2.00 [1.67–2.07] mg/L; *P* = 0.06) and was greater in nonbacteremic (2.45 [1.57–3.66] mg/L; *P* = 0.01) calves than healthy calves. No difference was detected between bacteremic and nonbacteremic calves (*P* = 0.32; Figure 2).

**Figure 1:**
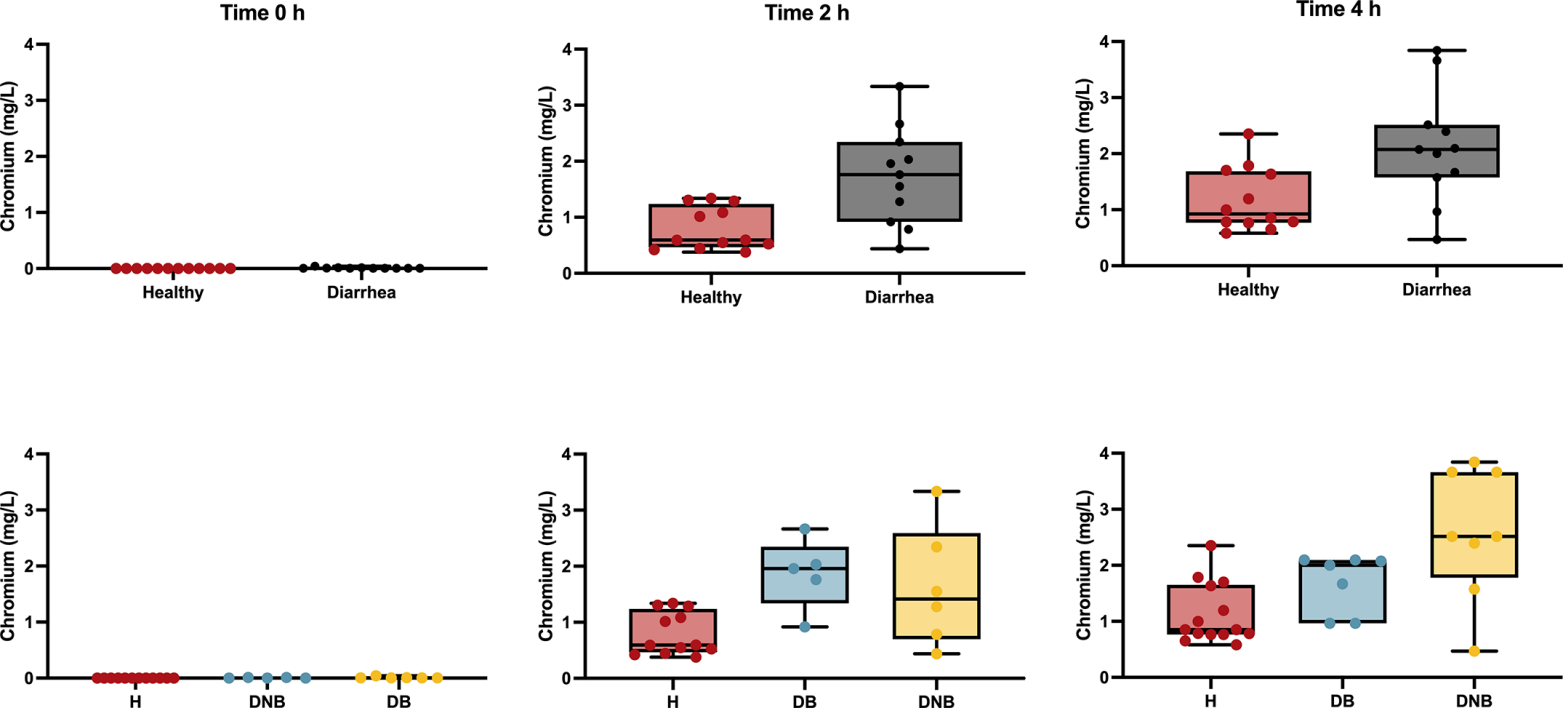
Plasma chromium (Cr) concentration at 0, 2, and 4 h after Cr-EDTA administration in healthy calves and calves with diarrhea. Diarrheic calves (n = 11) had greater median plasma Cr concentrations at 2 h (*P* = 0.007) and 4 h (*P* = 0.020) after Cr administration compared with healthy calves (n = 12). Plasma Cr concentration at 2 and 4 h after Cr-EDTA administration in healthy calves (H; n = 12), calves with diarrhea and bacteremia (DB; n = 5), and calves with diarrhea but no bacteremia (DNB; n = 6). Plasma Cr concentration at 2 h was greater in bacteremic (*P* = 0.004) or nonbacteremic (*P* = 0.04) calves compared with healthy, but no difference was detected between bacteremic and nonbacteremic calves (*P* = 0.20). Plasma Cr concentration at 4 h tended to be greater in bacteremic (*P* = 0.06) and was greater nonbacteremic (*P* = 0.01) calves when compared with healthy, and no difference was detected between bacteremic and nonbacteremic calves (*P* = 0.32). Box plots display the median and interquartile range, with whiskers extending to the minimum and maximum values. Individual observations are shown as dots.

**Figure 2:**
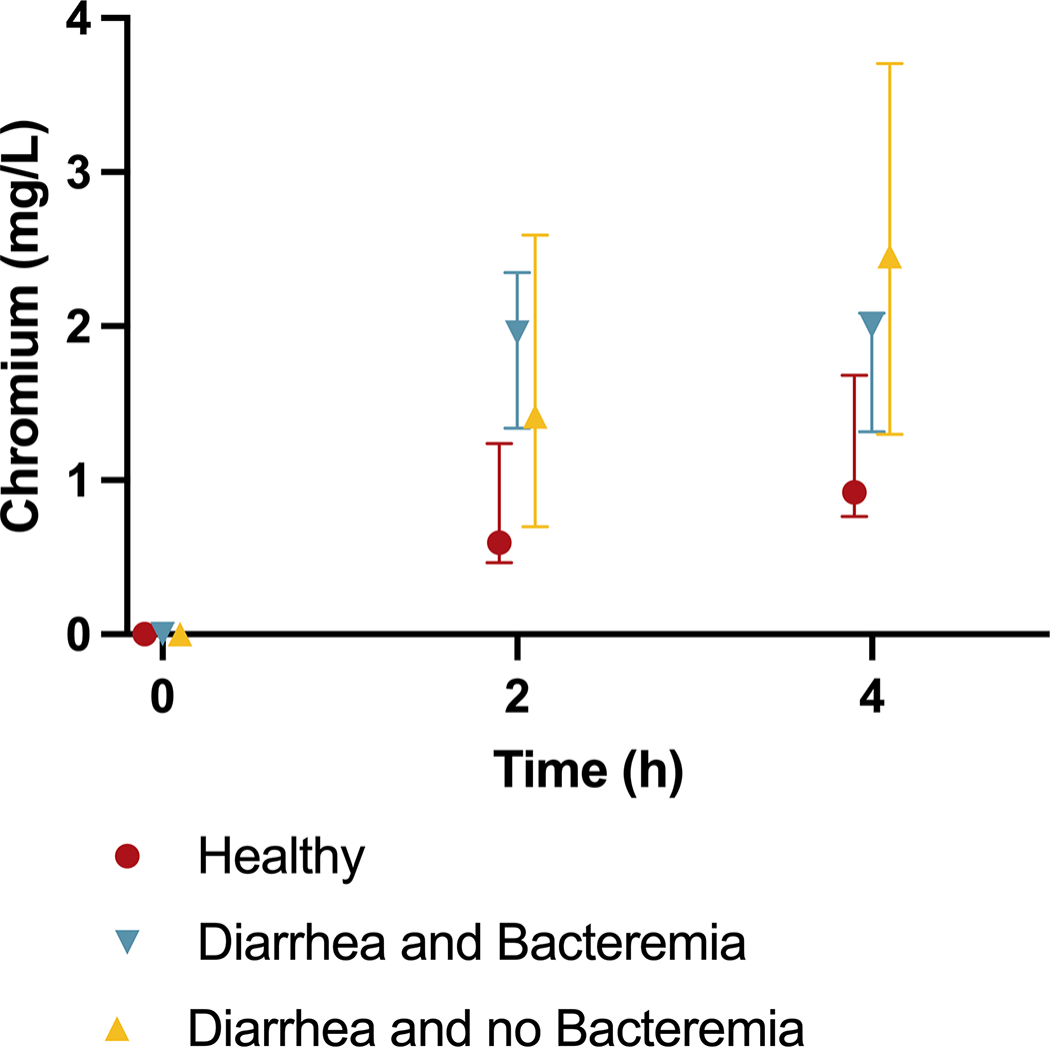
Plasma chromium (Cr) concentration at 2 and 4 h after Cr-EDTA administration in healthy calves (n = 12) and calves with diarrhea (n = 11) fed 2 L of milk replacer 2 h before Cr administration. The plasma Cr area under the curve calculated for 4 h (AUC_4_) for the healthy calves was 2.76 (95% CI: 1.24 to 4.28) mg/L × h, whereas the AUC_4_ for the diarrheic calves was 5.59 (95% CI: 2.49 to 8.69) mg/L × h. Among diarrheic calves, the AUC_4_ for the nonbacteremic (n = 6) was 5.66 (95% CI: 1.79 to 9.53) mg/L × h and 5.50 (95% CI: 3.52 to 7.48) mg/L × h for the bacteremic calves (n = 5). Error bars represent the median and interquartile range.

Plasma Cr concentration was lesser in healthy calves than in calves with diarrhea from which rotavirus A was identified in the feces (n = 6; 0.59 [0.48–1.19] mg/L vs. 1.42 [0.92–1.76] mg/L; *P* = 0.06), and in diarrheic calves from which both rotavirus A and *Cryptosporidium* spp. were identified in the feces (n = 4; 2.00 [1.37–2.68] mg/L; *P* = 0.009).

The plasma Cr AUC_4_ for healthy calves was 2.76 (95% CI: 1.24 to 4.28) mg/L × h, whereas the AUC_4_ for the diarrheic calves was 5.59 (95% CI: 2.49 to 8.69) mg/L × h. Among diarrheic calves, the AUC_4_ for the nonbacteremic was 5.66 (95% CI: 1.79 to 9.53) mg/L × h and 5.50 (95% CI: 3.52 to 7.48) mg/L × h for the bacteremic.

In the univariable linear regression models, heteroscedasticity was identified. Therefore, robust linear regression was performed. Healthy calves had lesser plasma Cr concentration (−0.94; 95% CI: −1.53 to −0.35 mg/L; *P* = 0.003) than diarrheic calves. Weight on arrival was not associated with Cr concentration (*P* = 0.43), and weight on arrival, plasma protein, hydration status, and room were not identified as confounders of the association of diarrhea status with plasma Cr concentration. Further, plasma protein concentration was not different between diarrheic and nondiarrheic calves (*P* = 0.10). Bacteremia status was not associated with Cr concentration in diarrheic calves (coefficient for positive blood culture: 0.25; 95% CI: −0.93 to 1.42 mg/L; *P* = 0.65).

Our findings indicate that neonatal calves with diarrhea have greater gut permeability soon after the onset of disease than healthy calves. Several factors probably contribute to this, including gut bacteria virulence factors and load, antimicrobial and anti-inflammatory drugs, and host factors, such as genetic predisposition to barrier leakiness (Fine et al., 2020). The mechanisms by which rotavirus and *Cryptosporidium* spp. affect intestinal permeability are not well understood (Navaneethan and Giannella, 2008); however, secondary intestinal inflammation, increased transit time, and potential dysbiosis may contribute to the gut permeability changes observed with these pathogens, and could be the explanation for why the calves positive for these pathogens in this study presented increased gut permeability. Additionally, dysbiosis itself may compromise the mucus layer, further increasing intestinal permeability (Di Vincenzo et al., 2024). Given that diarrhea is known to alter the fecal microbiota in calves (Gomez et al., 2017, 2022), future research could focus on exploring the role of gut microbiota in regulating intestinal permeability. As gut permeability was not accessed before and during diarrhea, a causal relationship between diarrhea and increased gut permeability could not be established.

No difference in plasma Cr concentration was detected between bacteremic and nonbacteremic diarrheic calves, suggesting a lack of difference in gut permeability. There are a few possible explanations for this lack of difference. Evaluating the plasma Cr difference between diarrheic bacteremic and nonbacteremic calves was not accounted for when the sample size calculation was performed. Therefore, we are probably underpowered for this comparison, possibly contributing to a type II error. Additionally, we only evaluated gut permeability using Cr, a marker of paracellular transport (Bischoff et al., 2014). Investigation of the gut barrier using bacteria-related assays, such as plasma D-lactate and lipopolysaccharide-binding protein, and biomarkers of intestinal inflammation, such as fecal calprotectin, may provide more comprehensive information regarding intestinal barrier function in diarrheic calves (Bischoff et al., 2014). Other markers of gut permeability that are frequently used in calves, such as mannitol and lactulose, have been shown to increase gastrointestinal transit time in humans (Read et al., 1982; Adkin et al., 1995). Therefore, we did not use them in this study, as the animals included likely already had decreased gastrointestinal motility due to diarrhea (Kirchner et al., 2015). The area under the plasma Cr concentration curve at time *t* depends on the absorption, distribution, metabolism, and excretion of Cr. We attributed differences in plasma Cr concentration over time primarily to increased intestinal absorption (increased intestinal permeability) because Cr is not metabolized and is highly bound to plasma proteins (Ducros, 1992), and hydration status and protein were not found to confound the relationship of diarrhea status with Cr concentration.

Blood culture is considered the gold standard diagnostic to detect bacteremia, but the sensitivity and specificity of this test are moderate (Hall and Lyman, 2006; Doern et al., 2019). Therefore, there is a chance of misclassification bias in this study. Collecting a larger volume of blood and blood from more than one site can improve the sensitivity and specificity of the test (Hall and Lyman, 2006; Doern et al., 2019). We aimed to avoid contamination during sampling and laboratory analysis; however, there is still a chance of contamination in our samples. It can be challenging to differentiate possible skin contamination from a true pathogen (Doern et al., 2019). Because we collected and processed the blood following the proposed guidelines (Doern et al., 2019) and collected the samples from sick animals, we believe our results are robust.

An important factor to consider when interpreting the results of this study is the abomasal emptying rate (**AER**). In healthy calves suckling 2 L of milk replacer, the AER as assessed by ultrasonography had a mean half-emptying time of 86 min (Marshall et al., 2008), whereas in diarrheic calves, AER is delayed (Kirchner et al., 2015). Based on a previous study that described differences in Cr concentration 2 h after administration (Pisoni et al., 2022), to account for possible delayed AER in diarrheic calves, we measured plasma Cr concentration 2 and 4 h after administration. We believe that with these 2 measurements in time we were able to characterize gut permeability of these calves. However, Figure 1 shows that mean plasma Cr was still increasing at 4 h; thus, measures for a longer time could have been informative, especially because Cr absorption kinetics have not been established in preweaning calves. Although milk and grain intake could have influenced the results of this study, calves consumed similar amounts of both. All calves drank all the morning milk and the median intake of grain in the week surrounding Cr administration was 25 g/d for both healthy calves (range: 0 to 136 g) and diarrheic calves (range: 0 to 78 g). Therefore, the impact of intake variation is likely minimal. Another factor that may have influenced our results is the inclusion of calves from 2 different rooms as both cases and controls, introducing the potential for group heterogeneity. Although room was not identified as a confounding factor in the linear regression model, suggesting its impact on our results was minimal, this possibility should still be acknowledged. Finally, an important limitation of our study is that the exact age of the calves is unknown. Although we selected cases and controls that had the onset of diarrhea on similar days after arrival at the facility, and BW was not found to be a confounder, there could have age-related differences in gut permeability that were not controlled for in this study.

This study suggests an association between diarrhea and increased gut permeability in neonatal calves. Further research is needed to investigate the relationship between gut permeability, bacteremia, and sepsis in neonatal calves with diarrhea.

## References

[bib1] Abuelo A., Cullens F., Brester J.L. (2021). Effect of preweaning disease on the reproductive performance and first-lactation milk production of heifers in a large dairy herd. J. Dairy Sci..

[bib2] Adkin D.A., Davis S., Sparrow R., Huckle P., Phillips A., Wilding I. (1995). The effects of pharmaceutical excipients on small intestinal transit. Br. J. Clin. Pharmacol..

[bib3] Araujo G., Yunta C., Terré M., Mereu A., Ipharraguerre I., Bach A. (2015). Intestinal permeability and incidence of diarrhea in newborn calves. J. Dairy Sci..

[bib4] Bischoff S.C., Barbara G., Buurman W., Ockhuizen T., Schulzke J.D., Serino M., Tilg H., Watson A., Wells J.M. (2014). Intestinal permeability - A new target for disease prevention and therapy. BMC Gastroenterol..

[bib5] Cangiano L.R., Villot C., Renaud J., Ipharraguerre I.R., McNeil B., DeVries T.J., Steele M.A. (2022). Induction of leaky gut by repeated intramuscular injections of indomethacin to preweaning Holstein calves. J. Dairy Sci..

[bib6] Constable P.D. (2004). Antimicrobial use in the treatment of calf diarrhea. J. Vet. Intern. Med..

[bib7] Corley L.D., Staley T.E., Bush L.J., Jones E.W. (1977). Influence of colostrum on transepithelial movement of *Escherichia coli* 055. J. Dairy Sci..

[bib8] Di Vincenzo F., Del Gaudio A., Petito V., Lopetuso L.R., Scaldaferri F. (2024). Gut microbiota, intestinal permeability, and systemic inflammation: A narrative review. Intern. Emerg. Med..

[bib9] Doern G.V., Carroll K.C., Diekema D.J., Garey K.W., Rupp M.E., Weinstein M.P., Sexton D.J. (2019). A comprehensive update on the problem of blood culture contamination and a discussion of methods for addressing the problem. Clin. Microbiol. Rev..

[bib10] Ducros V. (1992). Chromium metabolism - A literature review. Biol. Trace Elem. Res..

[bib11] Fine R.L., Manfredo Vieira S., Gilmore M.S., Kriegel M.A. (2020). Mechanisms and consequences of gut commensal translocation in chronic diseases. Gut Microbes.

[bib12] Gomez D.E., Arroyo L.G., Costa M.C., Viel L., Weese J.S. (2017). Characterization of the fecal bacterial microbiota of healthy and diarrheic dairy calves. J. Vet. Intern. Med..

[bib13] Gomez D.E., Li L., Goetz H., MacNicol J., Gamsjaeger L., Renaud D.L. (2022). Calf diarrhea is associated with a shift from obligated to facultative anaerobes and expansion of lactate-producing bacteria. Front. Vet. Sci..

[bib14] Hall K.K., Lyman J.A. (2006). Updated review of blood culture contamination. Clin. Microbiol. Rev..

[bib15] Kirchner D., Schwedhelm L., Wenge J., Steinhöfel I., Heinrich C., Coenen M., Bachmann L. (2015). Ultrasonographic imaging of abomasal milk clotting and abomasal diameter in healthy and diarrheic calves. Anim. Sci. J..

[bib16] Klein P., Kleinová T., Volek Z., Šimůnek J. (2008). Effect of *Cryptosporidium parvum* infection on the absorptive capacity and paracellular permeability of the small intestine in neonatal calves. Vet. Parasitol..

[bib17] Larson L.L., Owen F.G., Albright J.L., Appleman R.D., Lamb R.C., Muller L.D. (1977). Guidelines toward more uniformity in measuring and reporting calf experimental data. J. Dairy Sci..

[bib18] Marshall T.S., Constable P.D., Crochik S.S., Wittek T., Freeman D.E., Morin D.E. (2008). Effect of suckling an isotonic solution of sodium acetate, sodium bicarbonate, or sodium chloride on abomasal emptying rate and luminal pH in calves. Am. J. Vet. Res..

[bib19] Mohler V.L., Izzo M.M., House J.K. (2009). *Salmonella* in calves. Vet. Clin. North Am. Food Anim. Pract..

[bib20] Navaneethan U., Giannella R.A. (2008). Mechanisms of infectious diarrhea. Nat. Clin. Pract. Gastroenterol. Hepatol..

[bib21] Pisoni L., Devant M., Blanch M., Pastor J.J., Marti S. (2022). Simulation of feed restriction and fasting: Effects on animal recovery and gastrointestinal permeability in unweaned Angus-Holstein calves. J. Dairy Sci..

[bib22] Read N.W., Cammack J., Edwards C., Holgate A.M., Cann P.A., Brown C. (1982). Is the transit time of a meal through the small intestine related to the rate at which it leaves the stomach?. Gut.

[bib23] USDA (2021). Morbidity and Mortality in U. S. Preweaned Dairy Heifer Calves NAHMS Dairy 2014 Study Calf Component Information. https://www.aphis.usda.gov/sites/default/files/morb-mort-us-prewean-dairy-heifer-nahms-2014.pdf.

[bib24] Warren A.L., Stokol T., Hecker K.G., Nydam D.V. (2013). Storage-associated changes in the bovine hemogram with the ADVIA 120 hematology analyzer. Comp. Clin. Pathol..

[bib25] Zakia L.S., Gomez D.E., Constable P.D., LeBlanc S.J., Renaud D.L. (2023). Study protocol: Characterizing bacteremia in neonatal calves with diarrhea - A prospective cohort study. University of Guelph Libr. Atrium.

